# Disparities in Cardiovascular Disease Prevalence by Race and Ethnicity, Socioeconomic Status, Urbanicity, and Social Determinants of Health Among Medicare Beneficiaries With Diabetes

**DOI:** 10.5888/pcd22.240270

**Published:** 2025-03-06

**Authors:** Xilin Zhou, Joohyun Park, Deborah B. Rolka, Christopher Holliday, Daesung Choi, Ping Zhang

**Affiliations:** 1 Division of Diabetes Translation, National Center for Chronic Disease Prevention and Health Promotion, Centers for Disease Control and Prevention, Atlanta, Georgia

## Abstract

**Introduction:**

The association between various disparity factors and cardiovascular disease (CVD) prevalence among older US adults with diabetes has not been comprehensively explored. We examined disparities in CVD prevalence among Medicare beneficiaries with diabetes.

**Methods:**

Data were from the 2015–2019 Medicare Current Beneficiary Survey. Diabetes and CVD conditions — myocardial infarction (MI), stroke, and heart failure — were self-reported. We estimated the adjusted prevalence ratios (APRs) of CVD by race and ethnicity, education, income-to-poverty ratio (IPR), urbanicity, food insecurity, and social vulnerability using logistic regressions that controlled for these factors as well as age and sex.

**Results:**

Annually, an estimated 9.2 million Medicare beneficiaries aged 65 years or older had diabetes. Among them, 16.7% had MI, 13.7% had stroke, and 12.5% had heart failure. Beneficiaries who were food insecure, socially vulnerable, with an IPR less than or equal to 135%, and residing in rural areas had a higher crude CVD prevalence. After controlling for other factors, low IPR and food insecurity were linked to a higher prevalence of CVD. Hispanic beneficiaries had lower stroke and heart failure prevalence than non-Hispanic (NH) White and NH Black beneficiaries. NH Black beneficiaries had lower MI prevalence but higher heart failure prevalence compared with NH White beneficiaries. Female respondents with an IPR less than or equal to 135% had higher MI and stroke prevalence; this was not seen in male respondents.

**Conclusion:**

Low IPR and food insecurity were associated with higher MI, stroke, and heart failure prevalence among Medicare beneficiaries with diabetes. Our findings can inform targeted interventions to reduce CVD disparities in these populations.

**Summary**

**What is already known on this topic?**

Disparities in cardiovascular disease (CVD) prevalence are present in the general population and among people with diabetes.

**What is added by this report?**

We found that a low income-to-poverty ratio and food insecurity were positively associated with myocardial infarction, stroke, and heart failure among Medicare beneficiaries with diabetes. Disparities in CVD prevalence by race and ethnicity varied.

**What are the implications for public health practice?**

Our findings can assist with targeting intervention efforts toward people who are at an increased risk for CVD to reduce CVD disparities.

## Introduction

Cardiovascular disease (CVD) is among the leading causes of death among people with diabetes, accounting for approximately one-third of all deaths in this population in the US (1,2). However, the public health burden of CVD is distributed unevenly across groups. Research among populations with diabetes has identified factors such as low income, low educational attainment, and high social vulnerability as significant predictors of high CVD incidence, prevalence, and hospitalization and death rates (3–5). However, most existing studies have been conducted outside the US, making them less representative of the US adult population. The few US-based studies focused on a narrow set of disparity factors, highlighting the need for an updated analysis that examines a more comprehensive set of factors (2).

Moreover, research focusing on the older US population, who have a disproportionately higher prevalence of CVD compared with their younger counterparts, is lacking (6). One longitudinal study of adults aged 60 years or older with diabetes showed a consistently increasing hazard ratio for CVD-related death with each passing year (7). Given the increase in the number of older adults in the US population, the growing burden of diabetes will likely be accompanied by a corresponding increase in CVD cases (7).

Our study explored disparities in CVD prevalence among Medicare beneficiaries with diabetes based on factors including race and ethnicity, socioeconomic status, urbanicity, and social determinants of health (SDOH). Additionally, we conducted separate analyses by sex, given recent evidence of significant sex-specific differences in CVD prevalence, hospital admission rates, and death rates (8–10). Understanding CVD prevalence among different groups is crucial for developing effective treatment strategies to treat people with both CVD and diabetes, while also reducing disparities.

## Methods

### Data source and study sample

Data were from the Medicare Current Beneficiary Survey (MCBS), an annual survey of a nationally representative sample of Medicare beneficiaries in the US. This pooled cross-sectional analysis of the 2015–2019 MCBS focuses on Medicare beneficiaries aged 65 years or older with diabetes. The MCBS is sponsored by the Centers for Medicare & Medicaid Services (CMS) and is intended to monitor and evaluate Medicare programs by self-reported information on demographics, socioeconomic status, and health outcomes that are not captured in medical claims data. This study was exempt from the institutional review board’s review.

Diabetes was identified by an affirmative response to the question, “Has a doctor or other health professional ever told you that you had diabetes?” The outcomes of interest were self-reported myocardial infarction (MI), stroke, and heart failure. These 3 conditions were among the most frequently reported initial CVD complications in people with diabetes according to the Cardiovascular Disease Research Using Linked Bespoke Studies and Electronic Health Records cohort (11). Additionally, we created a composite variable to indicate if a beneficiary had any of the 3 CVD complications.

We examined the association between race and ethnicity (Hispanic, non-Hispanic [NH] White, NH Black, and NH Other), educational attainment (high school diploma or less vs more than high school diploma), income-to-poverty ratio (IPR [income ≤135% vs >135% of the federal poverty level]), urbanicity (rural vs urban), SDOH, and CVD prevalence. These factors are important markers of inequity, as identified in previous literature (12–15). We assessed 2 SDOH-related factors: food security (insecure vs secure) and social vulnerability (vulnerable vs not vulnerable). Food insecurity was a binary variable determined by using the US Department of Agriculture (USDA) Six-Item Short Form of the Food Security Survey module (16). The 6 questions are: 1) The food bought just didn’t last and I/we didn’t have money to get more; was that often, sometimes, or never true for you in the last 12 months? 2) I/we couldn’t afford to eat balanced meals; was this often, sometimes, or never true for you in the last 12 months? 3) In the past 12 months, did you ever cut the size of meals or skip meals because there wasn’t enough money for food? 4) How often did this happen? 5) In the past 12 months, did you ever eat less than you felt you should because there wasn’t enough money for food? 6) In the last 12 months, were you ever hungry but didn’t eat because there wasn’t enough money for food? If people gave positive responses (responses of often/sometimes or yes were coded as affirmative) to none or 1 of the 6 questions in the module, they were categorized as food secure (17). The USDA Food Security Survey Module is widely used to assess food insecurity, and studies have shown that it produces consistent results compared with other measures of food insecurity (18). Social vulnerability was a binary variable indicating whether the beneficiary’s county of residence ranked in the most vulnerable 20th percentile based on the Social Vulnerability Index. This index, created by the Centers for Disease Control and Prevention (CDC), assesses various social factors to determine the relative vulnerability of communities in their capacity to respond to hazardous public health events (19). It shows a strong association between high social vulnerability scores and worse health outcomes (19,20). In addition, we controlled for age group (aged 65–74 y and ≥75 y) and sex as confounding factors.

### Statistical analyses

We calculated the crude prevalence of MI, stroke, heart failure, and the composite of 1 or more of the conditions. For each CVD condition, we conducted logistic regressions to estimate the adjusted prevalence ratios (APRs) by each included factor (race and ethnicity, education, IPR, urbanicity of residence, food insecurity, and social vulnerability) (21). All factors except the one being examined, plus age group and sex, served as control variables in the regression models. We conducted separate analyses by sex for each CVD condition using the same statistical model. This separation was motivated by previous studies indicating significant sex differences in the response to the prevention of CVD and adverse CVD outcomes following a cardiac event (8–10). All estimates incorporated the sampling weights of MCBS and used the balanced repeated replication method of variance estimation in the pooled analysis (22). The weighted estimates represent the national noninstitutionalized population that was continuously enrolled in Medicare for at least 1 full calendar year during the study period. Year-fixed effects were also added in regressions to control for unobserved characteristics that change each year and are common to all beneficiaries for a given year. We report the estimates and their 95% CIs.

## Results

From 2015 to 2019, an estimated annual average of 9.2 million Medicare beneficiaries aged 65 years or older were living with diabetes (Table 1). Among them, 16.7% had MI, 13.7% had stroke, 12.5% had heart failure, and 32.2% had 1 or more of the 3 conditions. Overall, 58.7% of beneficiaries were aged 65 to 74 years; more than two-thirds were NH White (68.3%), and more than half had more than high school education (54.2%). Compared with male beneficiaries, female beneficiaries with diabetes tended to be older and included more NH Black beneficiaries. The female group also had lower educational attainment, had a higher percentage with an IPR of less than or equal to 135%, and were more likely to be food insecure.

**Table 1:** Sociodemographic Characteristics of Medicare Beneficiaries With Diabetes (≥65 y), Medicare Current Beneficiary Survey, 2015–2019^a^

Characteristic	Overall (N = 11,223)	Male (n = 5,520)	Female (n = 5,703)
Weighted average annual population	9,241,660	4,636,771	4,604,889
**Complications**			
Myocardial infarction	16.7 (15.6–17.7)	20.4 (18.6–22.2)	12.9 (11.5–14.4)
Stroke	13.7 (12.7–14.8)	13.5 (12.2–14.9)	13.9 (12.4–15.4)
Heart failure	12.5 (11.5–13.5)	12.0 (10.8–13.2)	13.0 (11.3–14.6)
Composite^b^	32.2 (30.8–33.5)	34.1 (32.1–36.2)	30.2 (28.0–32.5)
**Age group, y**			
65–74	58.7 (57.5–59.9)	60.6 (58.7–62.5)	56.8 (55.0–58.6)
≥75	41.3 (40.1–42.5)	39.4 (37.5–41.3)	43.2 (41.4–45.0)
**Race and ethnicity**			
Hispanic	10.2 (8.5–12.0)	9.4 (7.6–11.2)	11.1 (9.0–13.2)
Non-Hispanic White	68.3 (65.9–70.8)	71.5 (68.7–74.2)	65.2 (62.3–68.0)
Non-Hispanic Black	13.0 (11.8–14.2)	10.7 (9.2–12.2)	15.4 (13.5–17.3)
Non-Hispanic Other	8.4 (7.1–9.8)	8.4 (6.8–10.1)	8.4 (6.8–9.9)
**Education**			
High school diploma or less	45.8 (43.7–48.0)	39.9 (37.2–42.6)	51.9 (49.2–54.5)
More than high school diploma	54.2 (52.0–56.3)	60.1 (57.4–62.8)	48.1 (45.5–50.8)
**Income-to-poverty ratio^c^**			
≤135%	25.7 (24.4–27.1)	17.9 (16.4–19.4)	33.6 (31.7–35.6)
>135%	74.3 (72.9–75.6)	82.1 (80.6–83.6)	66.4 (64.4–68.3)
**Residence urbanicity**			
Rural	21.8 (20.2–23.3)	20.8 (19.1–22.5)	22.7 (20.5–24.9)
Urban	78.2 (76.7–79.8)	79.2 (77.5–80.9)	77.3 (75.1–79.5)
**Food insecurity**			
Food secure	91.8 (91.1–92.5)	94.5 (93.8–95.2)	89.1 (87.9–90.4)
Food insecure	8.2 (7.5–8.9)	5.5 (4.8–6.2)	10.9 (9.6–12.1)
**Social vulnerability^d^**			
Not vulnerable	81.0 (75.7–86.4)	82.0 (76.7–87.3)	80.1 (74.3–85.9)
Vulnerable	19.0 (13.6–24.3)	18.0 (12.7–23.3)	19.9 (14.1–25.7)

a Values are % (95% CI) unless otherwise indicated.

b The composite variable indicates that a beneficiary has any of the 3 conditions.

c Income-to-poverty ratio is defined as income less than or equal to 135% or greater than 135% of the federal poverty level.

d Social vulnerability indicates whether the beneficiary’s county of residence ranked in the most vulnerable 20th percentile based on the Centers for Disease Control and Prevention’s Social Vulnerability Index.

In terms of crude prevalence of CVD, heart failure was most prevalent among NH Black beneficiaries and those with lower educational achievement (Table 2). The prevalence of MI and stroke did not show significant differences by race and ethnicity and education. Stroke and heart failure were more prevalent among beneficiaries with lower IPR whereas MI was more prevalent among those residing in rural areas. All CVD conditions were more prevalent among beneficiaries experiencing food insecurity.

**Table 2:** Crude Prevalence of Cardiovascular Disease Among Medicare Beneficiaries With Diabetes, Medicare Current Beneficiary Survey, 2015–2019

Sociodemographic characteristic	Myocardial infarction	Stroke	Heart failure	Composite^a^
	% (95% CI)			
**Race and ethnicity**				
Hispanic	16.1 (13.5–18.7)	11.1 (8.7–13.5)	7.8 (5.3–10.3)	27.0 (23.8–30.1)
Non-Hispanic White	17.2 (16.0–18.5)	13.4 (12.1–14.7)	12.4 (11.1–13.7)	32.5 (30.7–34.4)
Non-Hispanic Black	14.4 (11.5–17.3)	16.4 (12.8–20.0)	18.6 (15.4–21.8)	35.3 (31.2–39.3)
Non-Hispanic Other	16.4 (12.4–20.3)	15.6 (12.0–19.3)	9.5 (6.1–12.9)	30.8 (25.6–36.0)
**Education**				
High school diploma or less	18.0 (16.3–19.7)	15.0 (13.6–16.4)	14.4 (12.7–16.1)	35.7 (33.9–37.6)
More than high school diploma	15.6 (14.2–17.0)	12.6 (11.2–14.1)	10.9 (9.6–12.1)	29.2 (27.4–30.9)
**Income-to-poverty ratio^b^**				
≤135%	19.1 (16.8–21.3)	16.6 (14.7–18.5)	15.9 (13.8–18.1)	37.2 (34.5–39.8)
>135%	15.9 (14.6–17.1)	12.7 (11.5–13.9)	11.3 (10.2–12.3)	30.4 (28.8–32.1)
**Residence urbanicity**				
Rural	20.5 (18.5–22.5)	15.6 (13.5–17.8)	14.8 (12.5–17.1)	36.9 (34.0–39.8)
Urban	15.6 (14.4–16.8)	13.2 (12.0–14.4)	11.8 (10.7–13.0)	30.9 (29.4–32.4)
**Food insecurity**				
Food secure	16.3 (15.2–17.3)	13.1 (12.1–14.1)	11.9 (11.0–12.8)	31.2 (29.9–32.6)
Food insecure	21.3 (17.6–25.1)	20.7 (16.9–24.5)	19.1 (14.9–23.2)	42.9 (38.4–47.3)
**Social vulnerability^c^**				
Not vulnerable	16.1 (14.9–17.2)	13.3 (12.1–14.5)	11.7 (10.7–12.7)	31.1 (29.6–32.5)
Vulnerable	19.3 (16.5–22.1)	15.5 (13.1–18.0)	15.8 (12.7–18.8)	36.9 (33.2–40.6)

a The composite variable indicates that a beneficiary has any of the 3 conditions.

b Income-to-poverty ratio is defined as income less than or equal to 135% or greater than 135% of the federal poverty level.

c Social vulnerability indicates whether the beneficiary’s county of residence ranked in the most vulnerable 20th percentile based on the Centers for Disease Control and Prevention’s Social Vulnerability Index.

Compared with White respondents, NH Black respondents had a lower prevalence of MI (APR = 0.80; 95% CI, 0.66–0.95) and a higher prevalence of heart failure (APR = 1.30; 95% CI, 1.02–1.58) (Table 3). Hispanic people had a lower prevalence of stroke and heart failure than both NH White people and NH Black people, with APRs ranging between 0.42 and 0.74. Beneficiaries with an IPR ≤135% had a higher prevalence of all CVD conditions than those with an IPR >135%, with APRs ranging between 1.16 and 1.26. In addition, beneficiaries residing in rural areas had a higher prevalence of MI than those in urban areas (APR = 1.25; 95% CI, 1.09–1.42). Beneficiaries experiencing food insecurity had a higher prevalence of all CVD conditions than those who were food secure, with APRs ranging between 1.37 and 1.53. No significant disparities were found based on social vulnerability.

**Table 3:** Adjusted Prevalence Ratios (APRs) of Cardiovascular Disease Among Medicare Beneficiaries With Diabetes, Medicare Current Beneficiary Survey, 2015–2019^a^

Sociodemographic characteristic	Myocardial infarction	Stroke	Heart failure	Composite^b^
	APR (95% CI)			
**Race and ethnicity**				
Hispanic vs NH White	0.87 (0.72–1.03)	0.74 (0.56–0.92)	0.55 (0.36–0.75)	0.76 (0.66–0.86)
NH Black vs NH White	0.80 (0.66–0.95)	1.11 (0.83–1.39)	1.30 (1.02–1.58)	1.01 (0.88–1.14)
NH Other vs NH White	0.91 (0.69–1.14)	1.11 (0.83–1.40)	0.73 (0.45–1.02)	0.92 (0.76–1.08)
Hispanic vs NH Black	1.09 (0.80–1.37)	0.67 (0.45–0.88)	0.42 (0.27–0.58)	0.75 (0.65–0.86)
Hispanic vs NH Other	0.96 (0.67–1.24)	0.67 (0.48–0.85)	0.75 (0.37–1.14)	0.83 (0.67–0.99)
NH Black vs NH Other	0.88 (0.62–1.14)	1.00 (0.69–1.30)	1.78 (1.08–2.48)	1.10 (0.90–1.30)
**Education**				
High school diploma or less vs more than high school diploma	1.11 (0.95–1.27)	1.10 (0.94–1.27)	1.17 (0.96–1.39)	1.16 (1.07–1.25)
**Income-to-poverty ratio^c^**				
≤135% vs >135%	1.25 (1.06–1.44)	1.20 (1.01–1.39)	1.26 (1.03–1.50)	1.16 (1.05–1.28)
**Residence urbanicity**				
Rural vs urban	1.25 (1.09–1.42)	1.13 (0.94–1.32)	1.13 (0.93–1.33)	1.12 (1.02–1.22)
**Food insecurity**				
Food insecure vs food secure	1.39 (1.13–1.64)	1.53 (1.24–1.82)	1.46 (1.11–1.82)	1.37 (1.20–1.53)
**Social vulnerability^d^**				
Vulnerable vs not vulnerable	1.16 (0.97–1.35)	1.08 (0.89–1.28)	1.21 (0.96–1.46)	1.13 (1.00–1.25)

Abbreviation: NH, non-Hispanic.

a Logistic regression models were used to estimate the adjusted prevalence ratios, adjusted for race/ethnicity, education, income-to-poverty ratio, urbanicity, food insecurity, and social vulnerability, in addition to age and sex.

b The composite variable indicates that a beneficiary has any of the 3 conditions.

c Income-to-poverty ratio is defined as income less than or equal to 135% or greater than 135% of the federal poverty level.

d Social vulnerability indicates whether the beneficiary’s county of residence ranked in the most vulnerable 20th percentile based on the Centers for Disease Control and Prevention’s Social Vulnerability Index.

We found different disparity patterns by sex (Table 4). Among male beneficiaries, those that were Hispanic had a lower prevalence of stroke and heart failure than both NH Black and NH White beneficiaries, with APRs ranging between 0.45 and 0.67. Also, among male beneficiaries, those that were NH Black had a lower prevalence of MI than NH White beneficiaries. We found no disparity by race and ethnicity in the prevalence of MI and stroke among female beneficiaries. Although no significant disparity in IPR was found in male beneficiaries, female beneficiaries with a lower IPR had a higher prevalence of MI, stroke, and the composite condition, with APRs ranging between 1.27 and 1.51.

**Table 4:** Adjusted Prevalence Ratios (APRs) of Cardiovascular Disease Among Medicare Beneficiaries With Diabetes, by Sex, Medicare Current Beneficiary Survey, 2015–2019^a^

Sociodemographic characteristic	Myocardial infarction	Stroke	Heart Failure	Composite^b^
	APR (95% CI)			
**Male sex**				
**Race and ethnicity**				
Hispanic vs NH White	0.81 (0.56–1.06)	0.67 (0.36–0.99)	0.52 (0.26–0.77)	0.74 (0.58–0.91)
NH Black vs NH White	0.67 (0.47–0.87)	1.24 (0.80–1.68)	1.14 (0.75–1.54)	0.98 (0.77–1.18)
NH Other vs NH White	0.82 (0.55–1.09)	1.21 (0.76–1.65)	0.51 (0.26–0.75)	0.90 (0.72–1.09)
Hispanic vs NH Black	1.20 (0.70–1.70)	0.54 (0.26–0.82)	0.45 (0.21–0.70)	0.76 (0.59–0.93)
Hispanic vs NH Other	0.99 (0.57–1.40)	0.56 (0.25–0.86)	1.02 (0.31–1.72)	0.82 (0.59–1.06)
NH Black vs NH Other	0.82 (0.49–1.16)	1.03 (0.52–1.54)	2.25 (1.15–3.35)	1.08 (0.79–1.37)
**Education**				
High school diploma or less vs more than high school diploma	1.11 (0.90–1.33)	1.29 (1.04–1.53)	1.25 (0.95–1.55)	1.16 (1.02–1.30)
**Income-to-poverty ratio^c^**				
≤135% vs >135%	1.05 (0.80–1.30)	1.07 (0.80–1.34)	1.21 (0.83–1.59)	1.04 (0.88–1.20)
**Residence urbanicity**				
Rural vs Urban	1.30 (1.09–1.50)	1.03 (0.83–1.24)	1.05 (0.73–1.36)	1.15 (1.02–1.27)
**Food insecurity**				
Food insecure vs food secure	1.37 (1.02–1.73)	1.19 (0.70–1.69)	1.74 (1.01–2.47)	1.32 (1.02–1.62)
**Social vulnerability^d^**				
Vulnerable vs not vulnerable	1.19 (0.88–1.50)	1.10 (0.77–1.42)	1.09 (0.76–1.43)	1.14 (0.95–1.32)
**Female sex**				
**Race and ethnicity**				
Hispanic vs NH White	1.00 (0.67–1.32)	0.80 (0.55–1.05)	0.59 (0.28–0.90)	0.78 (0.58–0.97)
NH Black vs NH White	0.99 (0.72–1.26)	1.02 (0.69–1.36)	1.41 (1.02–1.80)	1.04 (0.87–1.22)
Other vs NH White	1.11 (0.68–1.53)	1.03 (0.61–1.46)	0.96 (0.45–1.46)	0.94 (0.67–1.21)
Hispanic vs NH Black	1.00 (0.62–1.39)	0.78 (0.48–1.09)	0.42 (0.18–0.65)	0.74 (0.56–0.93)
Hispanic vs Other	0.90 (0.49–1.31)	0.78 (0.47–1.08)	0.61 (0.18–1.05)	0.83 (0.56–1.09)
NH Black vs Other	0.90 (0.51–1.29)	0.99 (0.57–1.41)	1.48 (0.68–2.27)	1.11 (0.80–1.42)
**Education**				
High school diploma or less vs more than high school diploma	1.12 (0.85–1.40)	0.96 (0.77–1.16)	1.13 (0.82–1.44)	1.17 (1.02–1.32)
**Income-to-poverty ratio^c^**				
≤135% vs >135%	1.51 (1.18–1.83)	1.30 (1.03–1.57)	1.28 (0.99–1.57)	1.27 (1.10–1.43)
**Residence urbanicity**				
Rural vs urban	1.18 (0.82–1.55)	1.21 (0.93–1.48)	1.20 (0.91–1.49)	1.10 (0.92–1.27)
**Food insecurity**				
Food insecure vs food secure	1.37 (1.01–1.74)	1.73 (1.32–2.14)	1.31 (0.97–1.66)	1.40 (1.21–1.59)
**Social vulnerability^d^**				
Vulnerable vs not vulnerable	1.10 (0.79–1.41)	1.08 (0.79–1.37)	1.29 (0.95–1.64)	1.11 (0.93–1.29)

Abbreviation: NH, non-Hispanic.

a Logistic regression models were used to estimate the adjusted prevalence ratios, adjusted for race/ethnicity, education, income-to-poverty ratio, urbanicity, food insecurity, and social vulnerability, in addition to age and sex.

b The composite variable indicates that a beneficiary has any of the 3 conditions.

c Income-to-poverty ratio is defined as income less than or equal to 135% or greater than 135% of the federal poverty level.

d Social vulnerability indicates whether the beneficiary’s county of residence ranked in the most vulnerable 20th percentile based on the Centers for Disease Control and Prevention’s Social Vulnerability Index.

## Discussion

Using data from 2015 to 2019, we found inverse associations between the prevalence of CVD and income-to-poverty ratio and food security status among a nationally representative sample of noninstitutionalized Medicare beneficiaries aged 65 years or older with diabetes. Those with a lower income level and with food insecurity had a higher prevalence of all 3 CVD conditions. In addition, we found that the relationship between race and ethnicity and CVD prevalence varied depending on the type of CVD; race and ethnicity exhibited a strong association with the prevalence of stroke and heart failure but a more modest association with the prevalence of MI. Such association was more often significant among male beneficiaries than female beneficiaries.

Our findings are generally in line with existing literature. Previous studies have consistently shown a higher prevalence of CVD among individuals in lower-resource groups, and similar associations have been observed with various factors in the general population, such as access to health care, the built environment, and social support (23–25). Among people with diabetes, studies have also documented associations between income, educational attainment, and cardiovascular outcomes (3–5). Our study offers a more comprehensive understanding of the differences in CVD prevalence among various groups. An adequately sized sample representing Medicare beneficiaries in the US strengthens the reliability and generalizability of the findings. We found that the prevalence of MI was higher among NH White people than NH Black people, while heart failure prevalence was higher among NH Black people than NH White people. Our findings align with previous studies showing a similar pattern in hospitalization rates for MI and heart failure (26,27). Moreover, while previous studies have documented differences in CVD prevalence between men and women, our study provides additional evidence showing that CVD prevalence also varies by disparity factor within each sex group.

Addressing disparities in complications and illnesses for people with diabetes is a priority because of its high prevalence, economic costs, and public health burden (28). Our study offers clear and comprehensive evidence on the factors associated with disparities in CVD prevalence. The findings can inform the development of CVD prevention interventions for people with diabetes, particularly by identifying relevant subpopulations to maximize the effectiveness of such interventions. The evidence from our study can help identify approaches to improving patient outcomes through nonmedical interventions.

Our study has several limitations. First, CVD conditions were self-reported, and the estimates only account for people who survived a CVD episode; this factor may result in an underestimation of the overall CVD disparity, as fatal CVD incidence may be more prevalent among disadvantaged populations (29). Similarly, diabetes was also self-reported, so people unaware of their condition were not included in the study, potentially introducing bias. According to the National Diabetes Statistics Report, 2.7 million people aged 65 years or older had undiagnosed diabetes in 2021 in the US (30). Second, as a cross-sectional study, our findings can only identify associations, not causality. Lastly, all potential confounders may not have been accounted for, which may have influenced the results.

A low IPR and food insecurity status were positively associated with the prevalence of MI, stroke, and heart failure. Our findings can help identify interventions to reduce CVD disparities among Medicare beneficiaries with diabetes in the US.
